# On-chip Microfluidic Multimodal Swimmer toward 3D Navigation

**DOI:** 10.1038/srep19041

**Published:** 2016-01-21

**Authors:** Antoine Barbot, Dominique Decanini, Gilgueng Hwang

**Affiliations:** 1 Laboratoire de Photonique et de Nanostructures, Centre National de la Recherche Scientifique, Marcoussis, 91460, France

## Abstract

Mobile microrobots have a promising future in various applications. These include targeted drug delivery, local measurement, biopsy or microassembly. Studying mobile microrobots inside microfluidics is an essential step towards such applications. But in this environment that was not designed for the robot, integration process and propulsion robustness still pose technological challenges. In this paper, we present a helical microrobot with three different motions, designed to achieve these goals. These motions are rolling, spintop motion and swimming. Through these multiple motions, microrobots are able to selectively integrate a chip through a microfluidic channel. This enables them to perform propulsion characterizations, 3D (Three Dimensional) maneuverability, particle cargo transport manipulation and exit from the chip. The microrobot selective integration inside microfluidics could lead to various *in-vitro* biologic or *in-vivo* biomedical applications.

Wireless mobile robots at the micro or nano-scale have promising applications in closed and liquid environments such as microfluidic channels or human blood vessels. Indeed, in such environments, installing transducers or sensors require for the moment invasive interventions. Different manners to propel such microrobots exist using electric^1^ and magnetic field^2^,^3^,^4^,^5^ or a propelling chemical reaction^6^,^7^. At this scale the Reynolds number is below 1 and therefore reciprocal motion is not an efficient way to propel an object^8^,^9^. Therefore a corkscrew form needs to be designed to transform a rotation in a propelling force as in a bacteria flagellum. Some milli-10,11,12, micro-13 and nanometric^14^ robots use this technique to efficiently propel with a rotation produced by the torque from a homogeneous magnetic field.

But even if such 3D controlled microrobots are promising for *in-vitro* or *in-vivo* applications, they have not yet been applied to such closed microfluidic environments. The main reason is their high surface-to-volume ratio, which is a handicap for robustness as they can be stuck on the substrate/channels walls or carry away by external flow (e.g. in a blood vessel). Indeed, we consider microrobots to be robust enough to be applied in such environments if they can be used for a long period of time without being damaged or lost during normal control.

A way of overcoming these problems is to make the robot capable of interacting with the surface. In fact either the friction directly created by the surface^15^ or the fluid viscous gradient caused by this surface^16^,^17^,^18^ are enough to propel a rotating microrobot. In this case the microrobot moves by rolling or tumbling on the surface. However its mobility is restrained in 2 Dimensions (2D) and high surface irregularities can become impassable obstacles for the microrobot.

The integration of microrobots on a microfluidic chip has two important consequences on the field. First it provides a well controllable environment in terms of flow and boundaries condition to investigate the physics of microrobots. Second it brings the microrobot to a challenging testbed. Indeed, to offer some practical applications, we think microrobots need to work in an environment designed for another purpose. Finally, the microfluidic chip is a common platform that requires manipulation and characterization at the micron scale. Microrobots could help fulfil this need.

This integration was demonstrated with milli-5, micro-19,20 and nanometric robots^21^ based on fluidic injection, manual integration or self-releasing technologies. The novelty of the proposed work is to present a selective self-integration of 3D helical swimmers inside microfluidic channels. We believe that self-integration is the most useful technique for selective microrobot integration with dimension less than 100 *μm*. Indeed at this scale injecting a single robot by fluidic inlet or by manual integration during the chip fabrication need a lot of technological development. However to support the drastic change of environment of the self-integration manoeuvre, both high robustness and mobility are required. Unfortunately, those cannot be provided by any single 3D corkscrew or surface motion independently. Therefore we investigate multimodal motion for microrobot as it increases the robustness and mobility by taking benefit of each motion advantages presented above while suffering less by their specific inconveniences. With this technique, we were able to perform self-integration of a microrobot in a microfluidic chip without fluidic injection or manual integration.

The Fig. 1a,b present the design and fabrication of the proposed multimodal helical microswimmer. We name this robot the Roll-To-Swim and refer to it in this article as RTS.

In this paper we first show how using three different motions enables us to integrate selectively the RTS in a microfluidic chip. The microfluidic chip is a well-controlled environment with minor fluid perturbation and it also simplifies the long term analysis of single microrobot. Therefore we use this platform to investigate the advantages and make a full characterization of each of the robot's motion. Finally we demonstrate useful microfluidic applications of multimodal motion which are: particle manipulation, 3D maneuverability inside a microfluidic chip and exit out of the chip.

## Microfluidic Chip Integration

Microfluidic chip integration of a microrobot is an important step. It proves the robustness of the RTS and brings helical robots closer to application and characterization by making them available on a widely used biological platform. In this section, we first describe the design and fabrication of the RTS and the microfluidic chip. Then we explain how we carried out this integration by using different motions.

**Figure 1:**
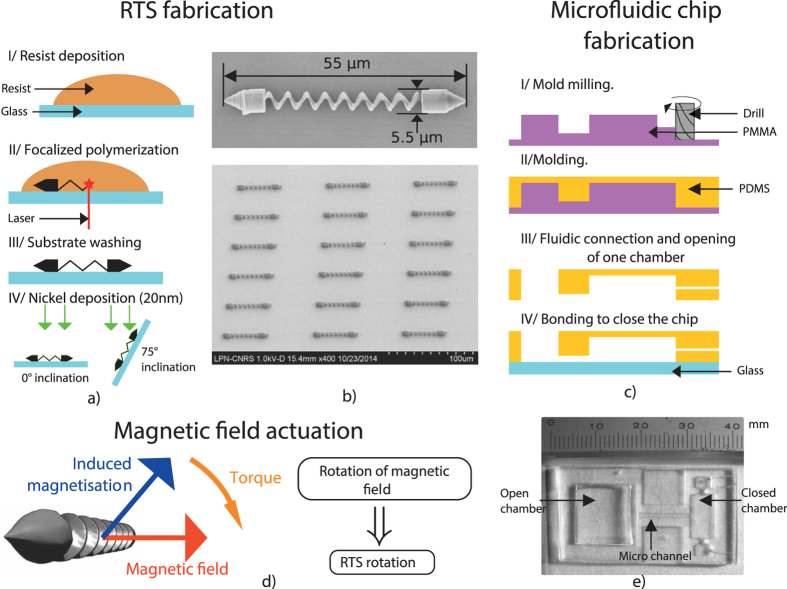
RTS and microfluidic chip fabrication description. (**a**) Different steps of the RTS fabrication. (**b**) SEM pictures of the RTS after all steps of fabrication have been completed and its dimensions. (**c**) Fabrication steps of the microfluidic chip. (**d**) Illustration of the actuation of RTS by a homogenous magnetic field. (**e**) Picture of the final chip. This field is produced by 3 orthogonal pairs of Helmholtz coils.

As shown in Fig. 1a,b The RTS is 55 *μ*m long for a 5 *μ*m diameter. Fabrication uses 3D two-photon laser lithography for patterning and e-beam nickel evaporation for ferromagnetic property. The method section presents more details on fabrication steps. A rotating homogeneous magnetic field produces the rotation of the robot, which is the base of the three motions. This is explained in Fig. 1d. The helical tail propels the robot with a corkscrew motion in the three dimensions by swimming in the fluid. Two conical heads provide interesting surface interaction. They allow both a rolling motion and a motion we refer as “spintop” where the robot spins on one head. Figure 1c,e display the fabrication steps of the microfluidic chip. It is made of glass and Polydimethylsiloxane (PDMS). There are two chambers connected by a microchannel. The first chamber is an open one to allow the RTS integration. The second one is a closed microfluidic chamber for the propulsion characterizations. A 50 *μ*m high, 1 mm wide and 11 mm long microchannel makes the connection between the two separate chambers.

**Figure 2:**
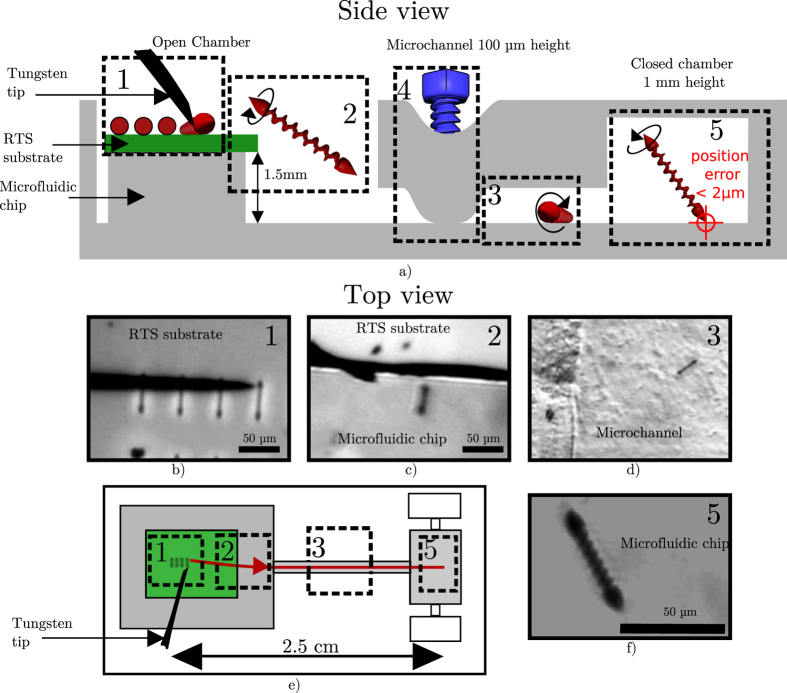
Integration of the RTS in the microfluidic chip. (**a**) Presents a side view of the chip with the different steps made to integrate the RTS on the chip. (**b**–**d**,**f**) are top-view photos of the experiments made by optical microscope. (**e**) is a top-view schematic of the chip. Supplementary video 1 displays a record of this integration.

For the integration process, we place in the open chamber of the chip a substrate with a field of RTSs on top of a thin PDMS layer. This substrate can be removed after the integration and used again to supply robots in different microfluidic chips. Figure 2 illustrates the five major steps to the integration of robots inside microfluidic chip. Supplementary video 1 shows a full record of a successful integration.

The first step is to detach a single RTS from the fabrication substrate (displayed in green in Fig. 2. The RTSs are fixed to the glass substrate during the fabrication process. A tungsten probe tip performs the detachment. It is piezo-actuated in 3 axes by the user control. Then we remove the tip and put a cover glass over the open chamber to limit the residual flow due to evaporation. Finally, the RTS uses the rolling motion to move to the edge of the substrate.

In the second step the RTS takes off the fabrication substrate and swims. Then it dives down around 1.5 mm to reach the bottom glass surface of the microfluidic chip. This distance corresponds to the thickness of the fabrication substrate of the RTSs and a thin PDMS layer. This PDMS layer helps to handle in an easier way the fabrication substrate. The taking off process is more difficult on nickel due to the high surface force. The conical design of the head is intended to ease this process. When the RTS lands on the bottom of the chip, it can use the rolling to move to the entrance of the micro channel.

In the third step, the RTS uses the rolling motion to move through the microchannel. The robustness to the flow of the rolling motion is needed here as some flux can remain between the two chambers. The reason for that is that the top of the open chamber only lays on the chip without permanent bonding. This can cause small evaporation which leads to the concentration of the residual flux in the microchannel. The fourth step consists of closing the microchannel. This is performed when the RTS reaches the characterization chamber. In that aim, a compressive pressure is applied with a screw pushing down the deformable PDMS top layer of the chip. After this step, the liquid is drained from the open chamber. The fabrication substrate is removed and stored for later use.

The final step illustrates that the spintop motion is used for precise motion inside the chip with single contact point to the surface. The position control is then limited by the resolution of our zoom optics. It is below 2 *μ*m as it is shown on the supplementary Fig. 3. To perform this integration process, the multimodal motions and the control ability of the RTS are essential. Both 3D maneuverability with swimming and surface motions are required.

## Characterizations of RTS Motions

**Figure 3:**
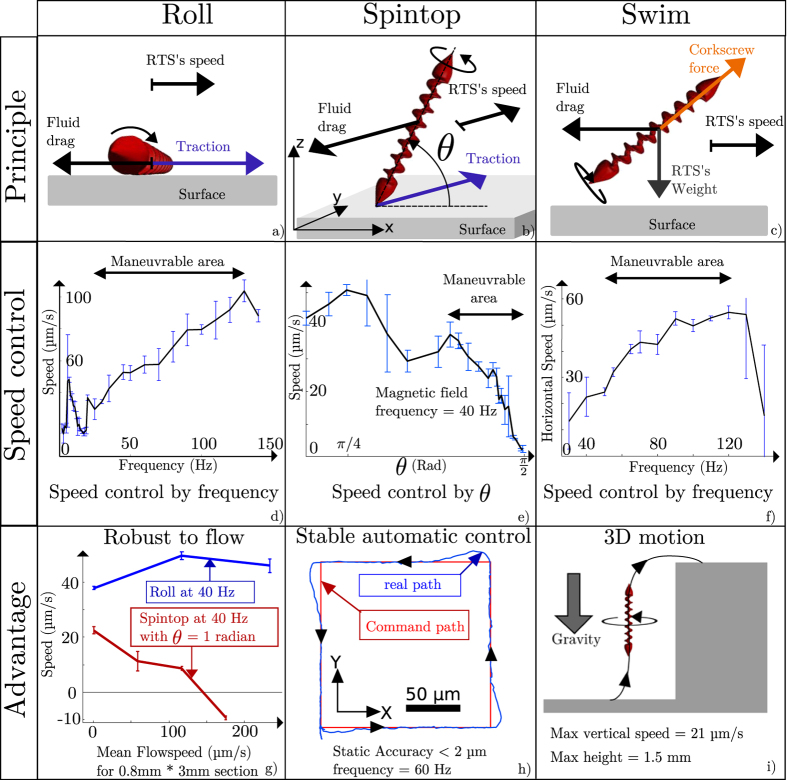
Presentation of the 3 possible motions of the RTS. A record of these motions is presented in Video 2. (**a**–**c**) Display the schematics of each motion. The displayed forces are the force applying on the RTS. The traction forces are due to the friction between the RTS and the surface. (**d**–**f**) Present the frequency domain and parameter of the actuations of RTS in each mode. For each point in the curves, four experiments are made on each side of a 200 *μm* square. Finally, (**g**,**h**) and (**i**) present the particular advantages of each motion. Video 3 shows a recording of the closed-loop control in spintop motion.

As mentioned in the introduction the microfluidic environment is well controlled in term of parasite flow and boundaries conditions. Therefore after the integration each RTS motion can be characterized separately in order to be better understood and controlled. For each motion, the Fig. 3 explains the force equilibrium, speed characterizations and main advantages of each motion. For the three motions, the rotation is around the main axis of the RTS. The inertia of the RTS is small so the transitional regime between different speeds is negligible. This is why we only consider cases where the sum of the forces applied on the RTS is zero and therefore the acceleration is null. For each characterization the average speeds on the four sides of a 200 *μ*m square trajectory were calculated. This reduces the perturbation induced by slightly tilted surfaces or parasite flows as effect in one direction balances the effect in the opposite direction. The error bars represent the standard deviation between the measurements on each side of the square trajectory. The point on the curves represents the mean value of the average speed on each of these sides.

While rolling, the RTS is in contact with the surface on all its length as illustrated on Fig. 3a. The RTS rotation creates a friction force with the surface that propels it. But depending on the surface friction, the robot can slide on the surface. Indeed, it advances less than its perimeter per each rotation. The supplementary Fig. 1 shows that this sliding depends on the surface material. The Fig. 3d shows the speed evolution versus the frequency of the magnetic field. Between 20 Hz and 140 Hz the surface friction increases proportionally with the magnetic field rotation. So the speed curve evolves in a linear way. Below 20 Hz the magnetization of the robot starts to change and aligns to an axis perpendicular to its length. This leads to a wobbling motion which increases the speed. Therefore the precise control of the robot is difficult as slow speeds are impossible. Through manual control, a control precision of about 20 *μ*m is achievable. Above 140 Hz the viscous drag is higher than the magnetic torque. This leads to a cut-off behaviour. In rolling motion, the RTS stays close to the surface. This has the advantage of being less influenced by the flow compared to the other motions. Figure 3g shows that rolling motions can move the RTS in an upstream flow. This is the great advantage of the rolling motion.

In spintop, the length of robot is not parallel to the surface any more. It touches the surface with the extremity of one head while spinning around its axis. To do this, the magnetic field direction rotates in a plane perpendicular to the surface. In this case the robot spins vertically and only the extremity of one head is in contact with the surface. Therefore the angle between the RTS length and the surface is 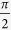 radians. This angle is the pitch angle. If this pitch angle decreases, the RTS is not vertical anymore and the integral of the friction force is no longer null. So the RTS starts to make a translational motion as shown on the Fig. 3b. The Fig. 3e displays the evolution of the speed with the pitch angle. The local surface interaction which varies on every experiment leads to an important standard deviation. Between 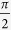 and 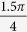 radians the speed decreases linearly to zero. This is achieved at a constant frequency (40 Hz) where there is no wobbling effect while speed control is made by the pitch angle. It is a key advantage compared to rolling because it makes spintop maneuverable at slow speed. This leads to the possibility of implementing a closed-loop control (Fig. 3h). It is carried out with a simple PID controller (Proportional Integral Derivative controller) that controls the speed through the pitch angle. A block diagram of this closed-loop control can be found on supplementary Fig. 2. This closed-loop control is essential to demonstrate the ability of precise positioning of the spintop motion. Indeed, the irregularity of the surface made manual positioning less precise. Moreover, the speed control of the RTS in spintop for an efficient positioning relies on user ability which made it hard to measure quantitatively. With the closed-loop control of spintop motion the static accuracy is below 2 *μ*m/s. Supplementary Fig. 3 shows more details on the precision of the control. To the best of our knowledge, the spintop motion is a newly reported motion of microrobots. Supplementary Fig. 4 shows that the shape of the head does not produce radical change on the propulsion characteristics. This lead us to believe that the surrounding fluid has a major role in the interaction between the head and the surface.

In the swimming motion, RTS is free from any surface. The rotation of the robot provides a corkscrew force from the helical part. This propelling force both compensates the gravity and propels the robot as explained on Fig. 3c. Just as rolling, wobbling happens at low frequencies. Because surface contact tends to suppress small wobbling. Therefore the frequency to avoid wobbling is above 40 Hz in swimming mode, as shown on Fig. 3f. The speed increases linearly with the magnetic field rotation frequency until 120 Hz. A cut-off behaviour also appears above this frequency. The great advantage of this mode is that it allows 3D control. The supplementary video 2 shows the RTS performs all three described motions. The supplementary video 3 shows a record of a closed-loop control of the RTS. Changing from one motion to the other is easy. The user just has to change the direction of the external magnetic field. Each motion gives the RTS a complementary advantage. It can be robust to the flow with the rolling motion. Precise and automatic control can be achieved in spintop. Finally, swimming motion allows 3D displacement.

## Microfluidic applications of the RTS

On top of the microfluidic chip integration, the multiple motions of the RTS allow other on-chip applications. The Fig. 3 shows three different examples of RTS’s maneuvres requiring multiple motions.

**Figure 4:**
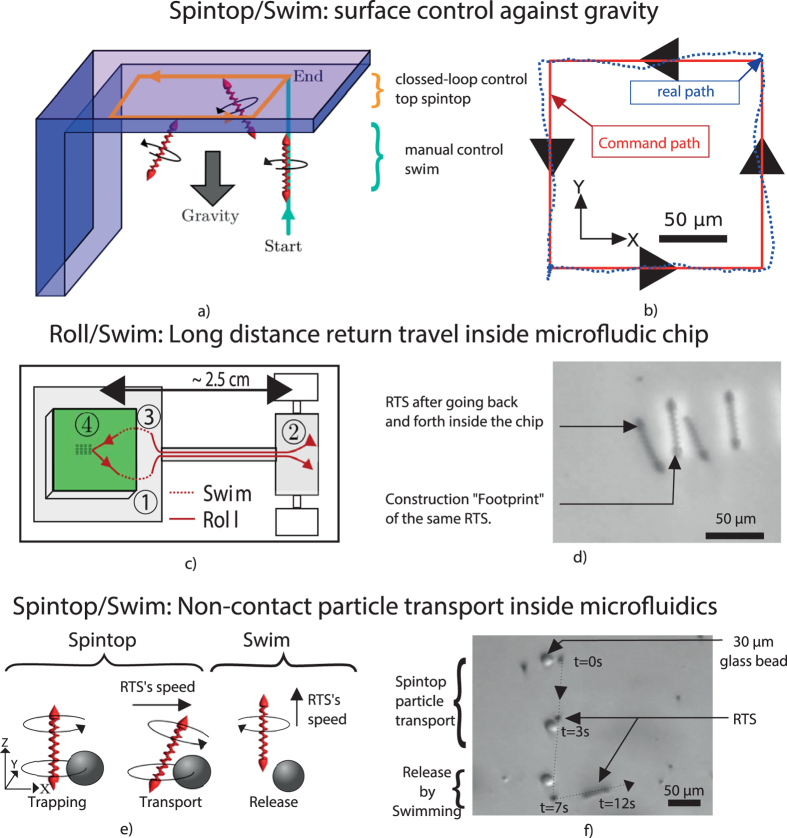
Different applications of combining motions. (**a**,**b**) and supplementary video 4 show how swimming can be used to go on a top surface and how spintop can control the RTS on this surface. (**c**,**d**) and supplementary video 5 shows that the RTS is able to go inside the microfluidic chip and comes back to its starting point on the fabrication substrate. (**e**,**f**) and supplementary video 6 show particle trapping, displacement by spintop motion and releasing of the particle by swimming.

The Fig. 4a shows a combination of swimming and spintop motion. First, the RTS can reach any surface by using the 3D swimming. Then, the spintop can displace the RTS on this surface even if the gravity force tends to detach it. In fact, the upward propelling force made by the corkscrew tail counterbalances the gravity. It provides enough friction for the spintop motion. The Fig. 4b shows that a closed-loop control similar to the one carried out on the bottom surface can be performed on the top surface of the chip. The performance of the closed-loop following this path is also similar even if the top cover is in PDMS and not in glass. The supplementary video 4 shows a record of these motion combinations of swimming and spintop.

The Fig. 4c shows an extension of the microfluidic chip integration. In this figure, we explain that long distance return travel is possible with the RTS. It shows that the RTS can be integrated inside the closed microfluidic channels but also be recovered from it. The operation follows the same step as the integration in the reverse order. However one difference is that the RTS is not diving from the fabrication substrate to the chip. Instead, it has to move up by swimming the 1.5 mm between the two. The Fig 4d shows that at the end the RTS came back around 10 *μ*m near the place it was initially detached. This ability could be useful to extract single object out of microfluidic channel. The supplementary video 5 shows a record of this return travel.

Finally, the Fig. 4e explains how the RTS combines spintop and swimming for cargo transport. This micromanipulation includes trapping, moving and releasing the particles inside the closed microfluidic channel. The Fig. 4e shows a schematic of these different steps. Spintop motion creates a vortex flow around the RTS. This vortex can trap adjacent particles of diameters up to 30 *μ*m. Then when the RTS moves the particle is still trapped and follows the robot by orbiting on a streamline around it. Supplementary Fig. 5 displays a two-dimensional numerical simulation of the moving RTS. To release the particle, the RTS switches to swimming motion. It moves up and the particle stays on the floor of the chip at a desired location due to the gravity force. The precision of this deposition is of around 10 *μ*m. The video 6 shows a record of the displacement of a particle through a microchannel.

## Discussion

This work was motivated by the hypothesis that providing multiple motions to a microrobot could significantly improve its robustness and mobility which are essential for its microfluidic applications. We believe that the selective self-integration of the RTS inside a microfluidic chip reported in this paper, validates this hypothesis. Indeed no major improvement on the system or on the robot fabrication was needed to achieve this integration. The capability of self-integration was not demonstrated yet to the best of our knowledge.

Moreover considering the microfluidic environment is well-controlled, we used it to characterize the propulsion behaviors of the three different motions availble in RTS. We also demonstrated that combining different types of motions added more functionalities such as particle cargo transport through microchannel, 3D navigation, in/exit out of a microfluidic device which are useful to further microfluidic applications.

The microfluidic chip integration not only proves the RTS capability but also constitutes a big step in itself toward future applications. Indeed it offers access to a controlled environment for sensitive physical characterizations and we plan to use this in the future to investigate the interactions between the robot, the fluid and the surface. It also provides a manipulation platform to make the microrobot interact with other elements such as, for example, particles, cells or antibodies. We expect that the demonstrated on-chip fluidic integrated microrobot will serve as an important platform, both to study micropropulsions and to test environments for various biological or biomedical applications.

## Method

### RTS’s fabrication

The RTSs consist of a polymer resist (IPG 780) and are made with a two-photon 3D laser lithography (Nanoscribe GmbH) using IPG 780 resist on a glass substrate. Metallization of 200 nm ferromagnetic layer (Ni) and 10 nm thick adhesion layer (Cr) were done by electron beam evaporation. Both 0 and 75 degrees of deposition inclination were used to make the RTS ferromagnetic in a more uniform way. The RTS is detached from the substrate with a tungsten tip fixed on a micromanipulator (kleindiek mm3A-EM).

### Magnetic Actuation setup

A pair of Helmholtz coils in each of the three space directions provide a quasi homogeneous 3D rotating magnetic field. These three pairs are linearly combined to provide a field in any wanted direction. Thus the ferromagnetic RTS aligns in this direction. The intensity of the field is constant and set to 12·10^−3^ Tesla. As it is impossible for dimension reasons to give the same size to each pair of coils, each one has different dimensions. However, the Helmholtz condition, which states that the radius of the two coils must be equal to the distance between the two coil centers, is respected. Therefore, the coils provide a homogeneous field at their center. The smallest dimension of the coil radius is 22.5 mm. The containing microfluidic chip is placed on a moving platform that is manually commanded in 3 translational axes. This keeps the RTS in a cube with 2 millimeters-long edges around the common center of the 3 pairs of coils and ensures that the RTS stays in a homogeneous region of the field.

### Control loop

In the manual control mode, the user can directly control the RTS with the keyboard. For that purpose, the arrow keys are mapped to the commands forward, backward, turn right or turn left. Four other keys are used to control the frequency of the rotating field and the pitch angle between the rotating magnetic field and the surface. The computer turns the command of the user into the corresponding changes in the parameters of the rotating field (angle and frequency). These changes are then updated in another thread which directly controls four linear amplifiers (Maxon motor 4-Q-DC) via a S626 card, with a refreshing time of 1 ms. Feedback is performed with a camera (Pike F032B) mounted on a microscope optic. The assembling of the camera and the optic allows a maximum precision of 0.5 micrometer per pixel. The image is sent back to the computer with a frequency of 20 frames per second to be displayed on the screen. Lighting of the sample can be made both by front light arriving through the optic or from back light depending if the RTS is on a opaque surface (nickel) or a transparent one (glass). Closed-loop control uses the same loop except that the video is also sent to a tracking algorithm that detects the robot position. Then a simple PID controller is used to control the RTS in position via the control of the pitch angle around 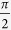 radians and update control position in the control thread. This control loop is summarized in the supplementary Fig. 2.

### Motion principle

When a ferromagnetic object is placed under a magnetic field, a resulting torque T is created. Its expression is :


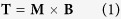


with **T** the torque, **M** the magnetization of the object and **B** the magnetic field.

The meaning of this equation is that the torque tends to align the magnetic axis with the magnetic field, just as a compass aligns with the magnetic field of the Earth. A rotating magnetic field will therefore make the object rotate at the same frequency. We used this rotation for the three motions of the RTS. However, this article will not focus on the primary axis of magnetization which is longitudinal to the RTS and result in a woobling. In fact, this axis makes the robot rotate around an axis that is perpendicular to its directions and is not used in the paper. To use only the second axis of magnetization, we increase the rotational frequency. Indeed, the rotation along the first axis of frequency reduces when the frequency rises. This is mainly due to a bigger increase of the drag force associated to the first axis of magnetization. Details of this phenomenon for a magnetic helical swimmer were described by Man *et al.*^22^.

### Microfluidic chip design

All experiments with the RTS were made inside a microfluidic chip after a self-integration of the robot. This allows a better control of parasite flow and provides a way of stocking the RTS with high yield reliability. The chip has 4 main parts:

- A first open chamber. It allows the introduction of the RTS fabrication substrate as well as the tip to remove a single RTS. This square chamber is large enough to allow manipulation with human hand precision and its surface is 1 *cm*^2^. When the fabrication substrate is put on this chamber, a thin PDMS layer is placed between this substrate and the chip. The first function of this layer is to prevent the fabrication substrate to slide when the robot is detached by the tungsten tip. The second is to facilitate the removal of this substrate by manually handed tweezers.
- A micro channel. It connects this chamber to a closed chamber. The section of this channel is a circular arc with a maximum height of 100 *μ*m and a width of 1 mm. The total length is 11 mm. The shape is designed to allow for the channel to be closed by applying a pressure on the top of the chip. Thus the closed part of the chip can be isolated from the exterior. A 2 mm diameter plastic screw is fixed just above the chip and can apply this compressive pressure.
- A closed experimental chamber. Its dimensions are 7 mm over 5 mm with a 850 *μ*m height. It is used for all the characterizations of the RTS. This chamber is also connected to a fluidic input and output. These connections can fill the chamber with liquid at the beginning and can be used to set a flow in the chamber to perform dynamics measurements or inject micro particles.
- An other microchannel. It is connected to the closed chamber and is used to store the RTS during the flushing period. Its dimensions are 50 *μ*m high for a width of 0.5 mm.

Another microfluidic chip, with two closed chambers instead of one, linked together by a 100 *μ*m high channel, has been used for the particle trapping and transport manipulation.

### Microfluidic chip fabrication

The chip is made with Polydimethylsiloxane (PDMS) and glass. First, a negative mold is made with a micro milling machine with a 0.5 mm diameter flat drill on Polymethyl methacrylate (PMMA). Then PDMS is poured on the mold and is put at least for 2 hours in a 60 °C oven in order for it to reticulate. Then the replicated PDMS channel is removed from the PMMA mold and bonded to a 1 mm thick glass substrate using an 0_2_ plasma (90 seconds at 70 Pa). Finally, the chip is put for 24 hours in a 60 °C oven to reticulate the PDMS entirely.

### Low Reynolds number approximation

All the experiments were carried out using isopropyl alcohol at 21 °C. The range of the maximum speed for the robots is below 300 *μm*·*s*^−1^. It corresponds to a Reynolds number inferior to 6.10^−3^ in isopropyl alcohol and therefore the laminar flow is guaranteed.

## Additional Information

**How to cite this article**: Barbot, A. *et al.* On-chip Microfluidic Multimodal Swimmer toward 3D Navigation. *Sci. Rep.* **6**, 19041; doi: 10.1038/srep19041 (2016).

## Supplementary Material

Supplementary video 1

Supplementary video 2

Supplementary video 3

Supplementary video 4

Supplementary video 5

Supplementary video 6

Supplementary Information
