# Supplementary material for: On-chip Microfluidic Multimodal Swimmer toward 3D Navigation
Source: Sci Rep. 2016 Jan 21;6:19041. doi: 10.1038/srep19041 (PMC4726319; doi:10.1038/srep19041)
Supplement: Supplementary Information [file srep19041-s7.pdf]

On-chip Microfluidic Multimodal Swimmer toward 3D Navigation – Supplementary information

Antoine Barbot[1], Dominique Decanini[1], and Gilgueng Hwang[1,*]

[*]gilgueng.hwang@lpn.cnrs.fr
[1]Laboratoire de Photonique et de Nanostructure, Centre National de la Recherche Scientifique, Marcoussis, 91460, France

ABSTRACT

This presents supplementary information and supplementary figure for the paper. Details on magnetic actuation, wobbling, numerical simulations of the field and of the flow are presented. The influence of the surface and of the head geometry on spintop motion are also shown.

1 Supplementary information

1.1 Videos from experiment

Several videos of the experiment were recorded. These videos were edited afterwards to change their speed and add supplementary information to help understanding.

The video 1 is a record of the self integration of the RTS inside the chip.

The video 2 demonstrates successively the three types of motions of the RTS. It also shows that the transition between these motions is straightforward.

The video 3 shows the robot following a square path by closed loop control. The closed-loop control is explained in details in Supplementary Fig. 2. The position error of the closed-loop is displayed in Supplementary Fig. 3

The video 4 illustrated the ability of the RTS to reach and move by closed loop control on a surface upside down. This means that the gravity tends to detach the robot from this surface.

The video 5 shows the ability of the RTS to go out of the microfluidic chip. In this video the robot come back to the fabrication substrate in the open chamber.

Finally the video 6 shows that the robot can trap a particle of size around 10 μm and move it through a micro channel of 100 μm height.

1.2 Magnetic actuation principle

The homogeneous field is produced by three pairs of Helmholtz coils. Each of the pairs aligns in one direction of the space. The Helmholtz condition guarantees the homogeneity of the field. However this condition only states that the derivative of the field intensity along the symmetrical axis is null in the middle of the two coils. Moreover our coils are not circle ideal coils as states the Helmholtz condition. For this reason we perform numerical simulation to quantify the non-uniformity of the field around the center. The simulations were made by the software Comsol for each coil pair. The results are presented in the supplementary figure 6. It shows that in a cube of 2 mm side the intensity of the flow varies less than 0.1 %.

1.3 Wobbling phenomena, influence on each mode

Wobbling of helical artificial swimmers in low Reynolds fluid environment has been described experimentally.[1] This phenomenon is due to the helical geometry of the swimmers. If the drag force is integrated along a rotating helix, a torque perpendicular to the axis of the helix appears. Even if the average of this torque during one revolution of the helix is zero, it is not null at given moment. This oscillating torque leads to the wobbling of the RTS at low frequency. Indeed when increasing frequency the fluid drag which counterbalances the oscillating rotation, increases much more than the one associated with the corkscrew rotation. This drag acts like a stabilization torque and the wobbling tends to become zero when the frequency increases. To be more precise, the Mason number which represents the ratio between the viscosity and the magnetic torque, is used. This number evolves between 0 and 1. The wobbling angle is proportional to the inverse of the mason number. The authors refer to the work of Man *et al.*[2] for details calculation of the force on a rotating helix and about the Mason number.

However, in the RTS case the magnetization is not made by a permanent magnet with a constant magnetization angle but by a ferromagnetic layer of nickel. Therefore when the external magnetic field is not aligned with the helical tails the

magnetization of the RTS will gain a component on the perpendicular axis of the tails. This will lead the robot to wobble even more. Moreover a residual magnetization exists and therefore the behaviour of the robot can depend on its previous conditions.

We do not yet control well this residual magnetization and the part it has in the wobbling phenomena. This is why we prefer to avoid low frequency region for the multiple motions of our robots. It is also why we didn't choose to use a tumbling motion even if the robot could do such motion at low frequency. In fact the transition between a tumbling and a wobble rolling was depending too much on previous condition. Thus stability of such motion was too complicated for us to to characterise and to model.

After this explanation we want to stress again the importance of the spintop motion, which is to our knowledge a newly introduced motion for microrobots. This motion allows us to reach continuously a null speed while remaining at a constant high frequency and therefore avoiding the wobbling problem. It is a simple way for the helical micro robots to get precise localization and for this reason we believe this motion is very promising.

1.4 Numeric simulation : particle trapping

The Fig. 5 presents a simple simulation which explains how the trapping of a particles is possible by spintop motion. The flow is calculated in a cut plane parallel to the surface. A rotating disk is representing the section of the RTS. An inlet flow speed is set to represent the motion of the robot. As we perform spintop at small speed we make the approximation that the robot is standing perpendicularly to the surface. Due to the strong hypothesis of the model, we do not pretend to give quantitative results. However we can see that the rotation of the robot makes the streamlines of the generated flow around it become closed. Therefore if a particle is on one of this streamline, it will be trapped by the RTS.

1.5 Surface influence, Nickel versus glass

From the extended Fig. 1 it is clear that the substrate material has a great influence on the motion by changing the friction coefficient between the robot and the surface.

2 Supplementary data figure

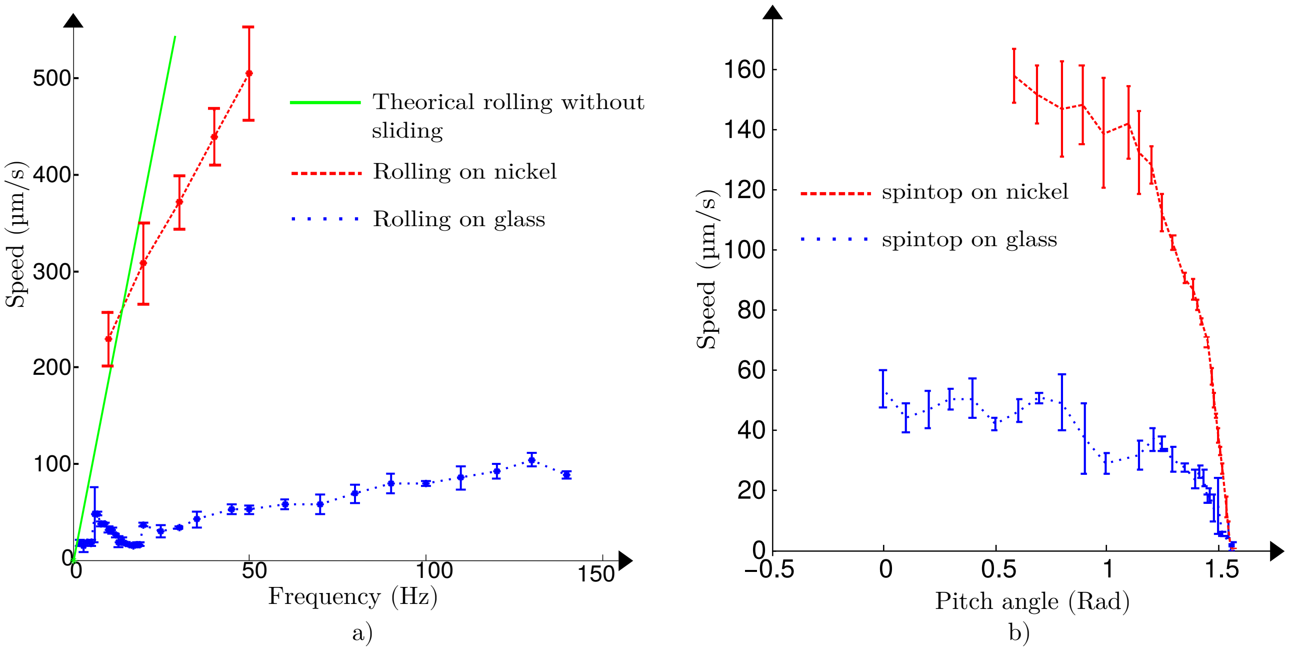

Figure 1. Influence of substrate material for surface base motion. a) shows that rolling motion is much faster on a nickel substrate than on glass as the RTS tends to slide less. At 10 Hz on nickel the robot starts to wobble allowing it to get a higher speed than theoretical one. b) shows that spintop is also faster on nickel substrate for the same reason. Each speed was measured four times on the same RTS

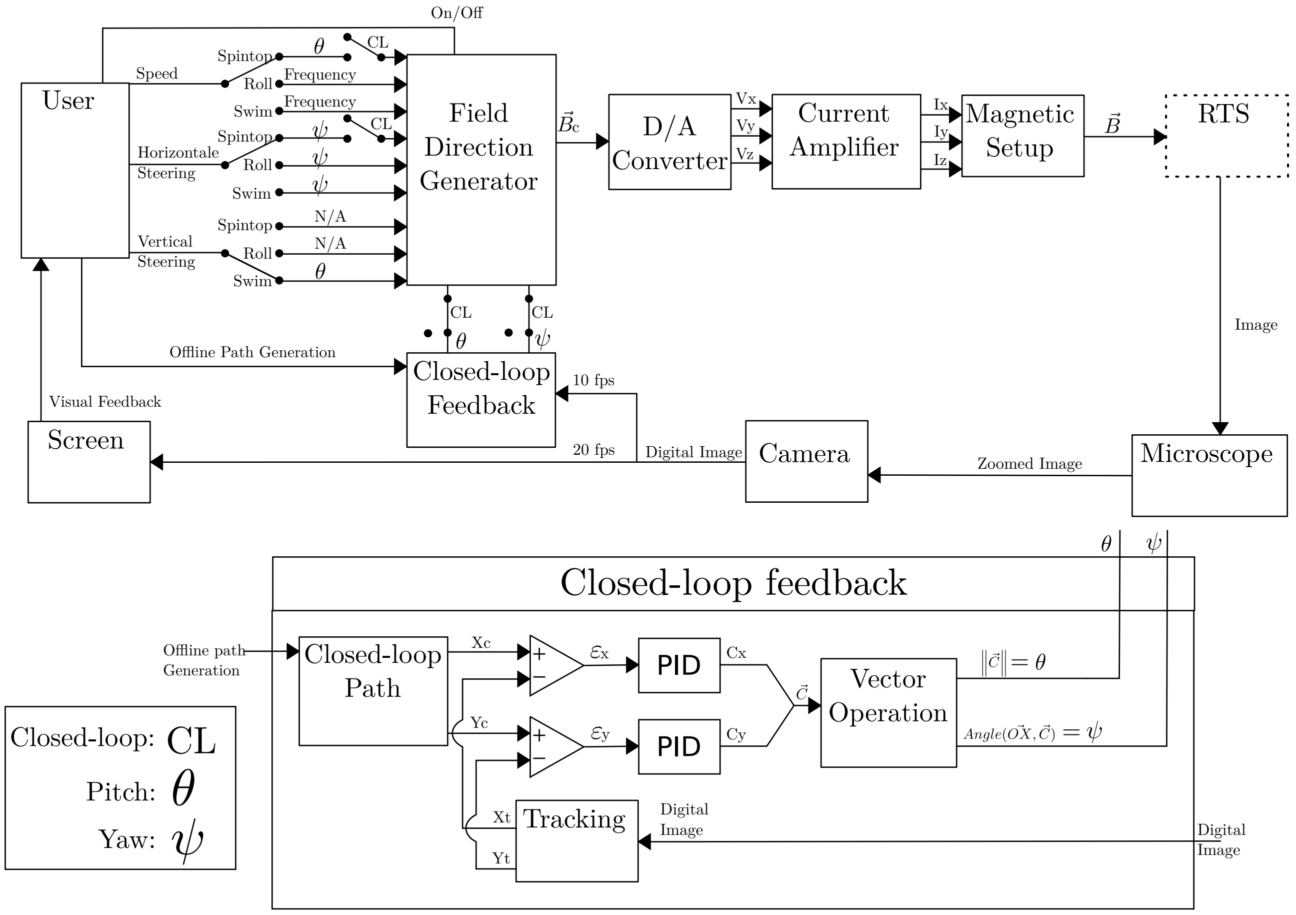

Figure 2. Control block diagram of the RTS. During open-loop, the user can control three parameters : Speed, horizontal and vertical steering. Depending on the motion these parameters are led by different parameters as explained in the Fig 2 of the main article. The ”CL” switches show how the command are mapped differently in the closed-loop configuration. The switches are represented in the closed-loop case which is possible only for spintop motion.

References

1. Peyer, K. E., Zhang, L., Kratochvil, B. E. & Nelson, B. J. Non-ideal swimming of artificial bacterial flagella near a surface. *Proceedings - IEEE Int. Conf. Robot.* 96–101 (2010).
2. Man, Y., Lauga, E. & Introduction, I. The wobbling-to-swimming transition of rotated helices. *Phys. Fluids* **071904**, 1–16 (2013).
3. Hecht, F. New development in freefem++. *J. Numer. Math.* **20**, 251–265 (2012).

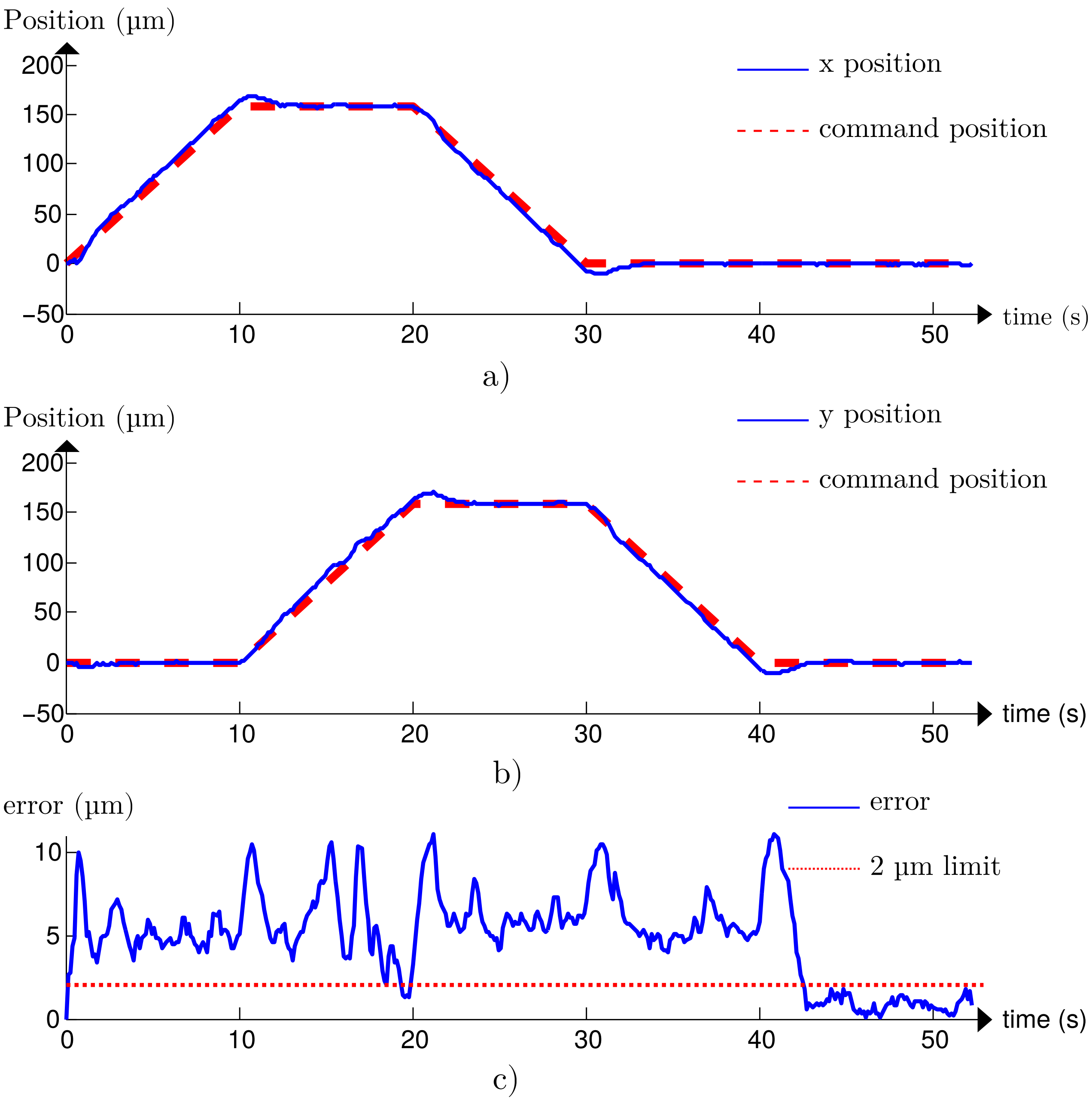

Figure 3. Accuracy of the spintop during closed-loop control. Both the command path and track path are displayed for a 150 μm square trajectory. a) shows the error in X positioning, b) in Y positioning. c) show the distance between the current position and the control one with the time. this error is below 2 μm for a fix command.

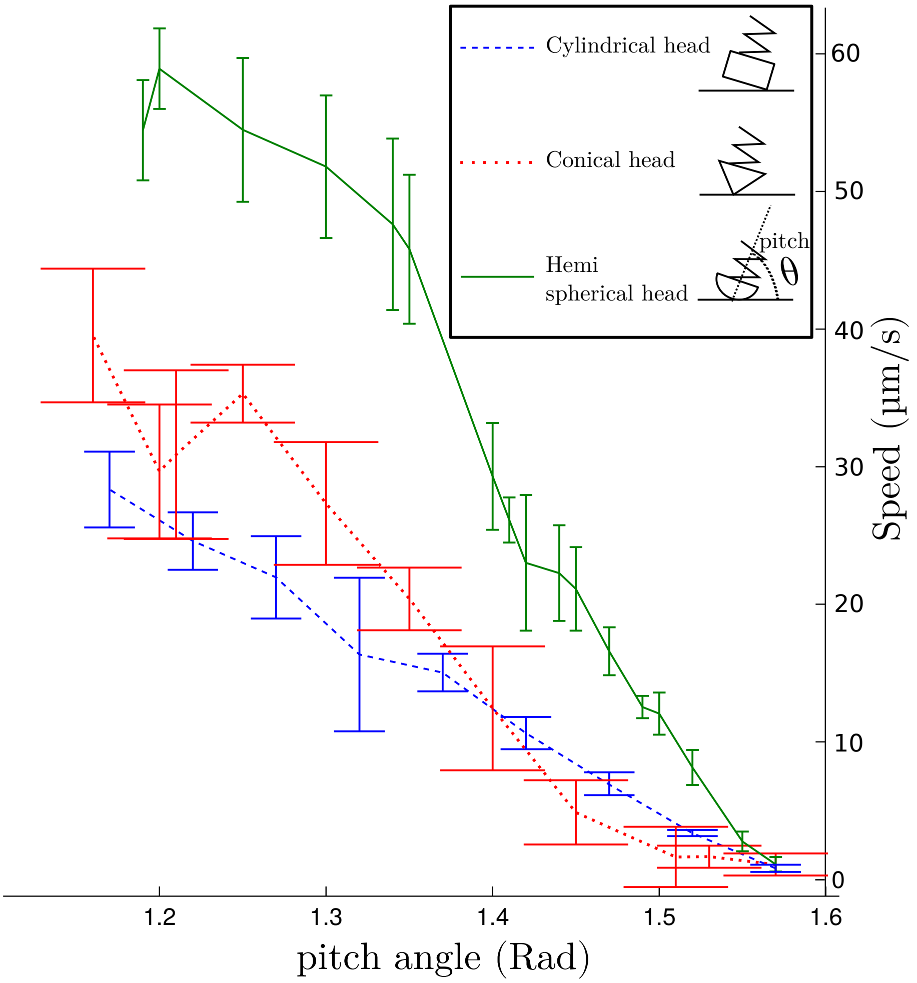

Figure 4. Spintop speed characterizations for different head designs. The frequency was set to 60 Hz for all experiments. For each characterisation the average speeds on the four sides of a 200 μm square trajectory were calculated. Therefore, the error bar represents the differences of speed on each directions due to remaining flow and small tilting of the surface. For a $\frac{\pi}{2}$ radian pitch angle the measured speed is slightly superior to zero due to residual flow in the chip and vibration of the camera. The relative similarity of the curve for different head geometries leads us to believe that a friction model base on a single contact point is here irrelevant. The main hypothesis is that, at least on glass surface, the fluid plays an important role on the friction between the surface and the robot. As the head shape does not influence greatly the speed of the robot, conical head was chosen to reduce the surface interaction during the take-off process on nickel. Indeed nickel is a sticky surface for the robot. Take off is eased by choosing the conical head.

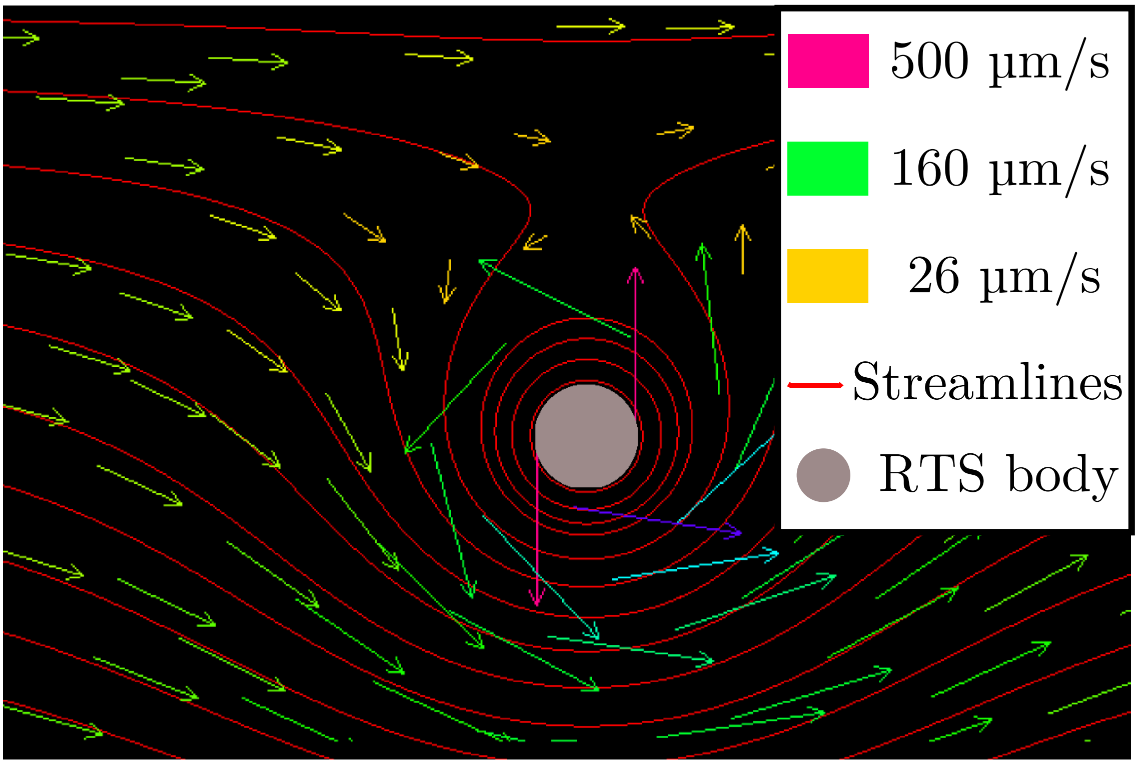

Figure 5. 2D Fluid Numerical simulation. The moving robot is simulated by a rotating cylinder. Flow stream velocity = $100\mu m \cdot s^{-1}$ and the rotation speed is $60Hz$. A particle located in this streamlines will be trapped around the RTS. The simulation are made with FreeFem++ software.[3]

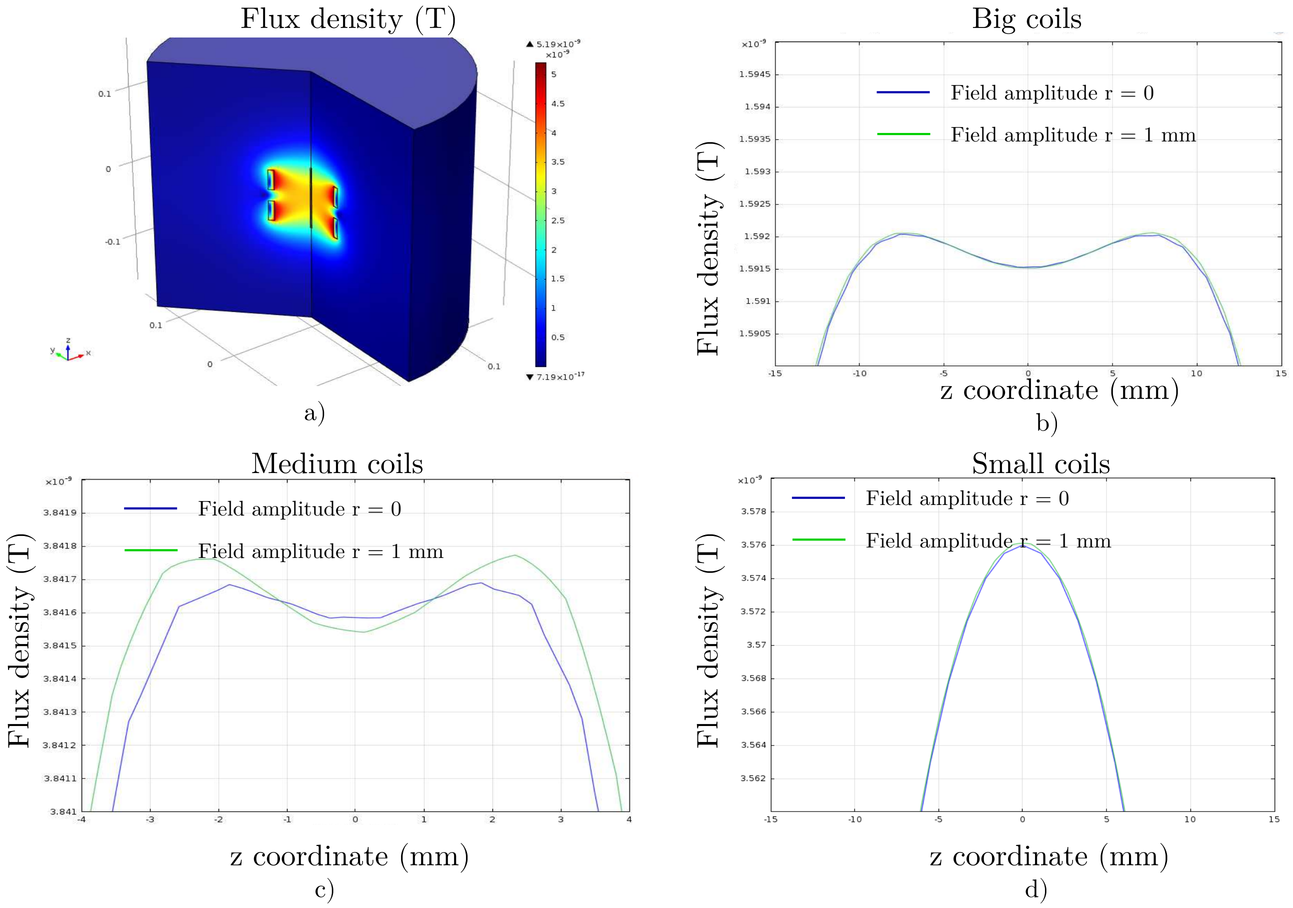

Figure 6. Numerical simulation of the magnetic field homogeneity around the center. b), c) and d) are respectively the large, medium and small pairs of Helmholtz coils. The variation is represented along the symmetrical axis and along a parallel axis at a distance of 1 mm. The difference of intensity of the field is less than 0.1 % in a 2 millimetres edge cube around the center
